# A Cognitive Ethology Study of First- and Third-Person Perspectives

**DOI:** 10.1371/journal.pone.0092696

**Published:** 2014-03-26

**Authors:** Joseph D. Chisholm, Craig S. Chapman, Marvin Amm, Walter F. Bischof, Dan Smilek, Alan Kingstone

**Affiliations:** 1 Department of Psychology, University of British Columbia, Vancouver, British Columbia, Canada; 2 Faculty of Physical Education and Recreation, University of Alberta, Edmonton, Alberta, Canada; 3 School of Psychology, University of Aberdeen, Aberdeen, United Kingdom; 4 Department of Computing Science, University of Alberta, Edmonton, Alberta, Canada; 5 Department of Psychology, University of Waterloo, Waterloo, Ontario, Canada; University of Tokyo, Japan

## Abstract

The aim of the present study was to test the cognitive ethology approach, which seeks to link cognitions and behaviours as they operate in everyday life with those studied in controlled lab-based investigations. Our test bed was the understanding of first-person and third-person perspectives, which in lab-based investigations have been defined in a diverse and multi-faceted manner. We hypothesized that because these lab-based investigations seek to connect with how first- and third-person perspective operates in everyday life, then either some of the divergent lab-based definitions are missing their mark or the everyday conceptualization of first- and third-person perspective is multi-faceted. Our investigation revealed the latter. By applying a cognitive ethology approach we were able to determine that a) peoples’ everyday understanding of perspective is diverse yet reliable, and b) a lab-based investigation that applies these diverse understandings in a controlled setting can accurately predict how people will perform. These findings provide a ‘proof of concept’ for the cognitive ethology approach. Moreover, the present data demonstrate that previous lab-based studies, that often had very different understandings of first- and third-person perspective, were each in and of themselves valid. That is, each is capturing part of a broader understanding of perspective in everyday life. Our results also revealed a novel social factor not included in traditional conceptualizations of first-person third-perspective, that of eye gaze, i.e., eye contact is equated strongly with first-person perspective and the lack of eye-contact with third-person perspective.

## Introduction

For decades experimental psychology in general, and cognitive psychology in particular, has benefited from the rigorous application of a scientific research approach based on *simplifying* and *controlling* the experimental situation in order to discover causal relationships between factors, such as the relation between levels of stimulus processing and memory for those processed items [1] or attentional selection and visual masking as evidenced in the “attentional blink” phenomenon [2]. Though this methodological approach has been extremely successful in generating reliable effects within a laboratory setting, it has also exposed profound limitations. While the intent was that an empirical foundation would be created that enabled researchers to develop cognitive constructs and theories that were universally valid, the reality is that usually the relationship between two factors is reliable if, and only if, particular environmental contexts and controls are present. Change the context or remove the controls and the relationship between factors becomes unpredictable. For example, what people remember depends not just on the level that a stimulus is processed but on whether the environment during encoding is the same as during recall (e.g., [3]). In a similar vein, what is visually selected or masked does not depend merely on the stimuli that are presented but on what items are expected to be seen (e.g., [4]).

The fact that cognitive processes change with the situational context in which a subject is embedded presents a serious challenge to researchers. Many have chosen to redefine the scope of their research objectives to understanding how a particular phenomenon, such as inhibition of return, behaves only in a laboratory setting [5], whereas some have emphasized the importance of investigating behaviour that emerges within more natural contexts, typically involving a complex analysis of coordinated sequences of actions [6]–[10].

In addition to these approaches, others have begun to embrace the fact that cognition varies with situational context and have turned it into the very focus of their laboratory enterprise, as is the case with embodied and distributed cognition [11]–[15]. An early response to this context-based challenge, exemplified by the celebrated cognitive scientists Donald Broadbent and Ulric Neisser, has been to work tirelessly to make scientist aware that their work risks having little relevance to real life if their effects are constrained to changes that occur within the laboratory [16] [17]. Broadbent and Neisser’s challenge is for researchers to discover a way to establish a valid empirical link between the work that they do in the lab to everyday phenomena.

In response to the call of Broadbent and Neisser, a research approach called Cognitive Ethology was advanced by Kingstone, Smilek, and colleagues [18]–[21]. Although the cognitive ethology approach shares elements with other studies that emphasize the need to understand behaviour from within more natural contexts [6], [8] the aim was to provide the research scientist with a methodology for bridging the gap that exists between uncontrolled real-life phenomena and controlled laboratory investigation, so that the effect studied in the lab can make direct and relevant contact with everyday life. In a nutshell, cognitive ethology proposes that one should ideally study a phenomenon first as it naturally occurs within a complex real-world environment *before* trying to move its investigation into more simplified and controlled lab-based situations. By starting at the natural level, one’s subsequent investigations are grounded in cognition and performance as it occurs in real life, and hence, through comparison between life and lab, one can determine which lab-based findings are likely to scale up to a natural environment and which findings are specific to a controlled research environment.

In the past few years, a great deal of published work has confirmed cognitive ethology’s underlying assumption that the way people behave in natural situations, and the conclusions that one draws from that behaviour, are often very different from the behaviour and conclusions generated in the lab. For example, lab-based research has demonstrated that when people are presented with images containing people, their attention is drawn automatically toward their faces and eyes [22]–[25]. Yet, recent investigations have demonstrated that this effect is often reversed in a natural social situation, such that people avoid looking at other people in the face and eyes when there is a potential for social interaction [26]–[29]. For example, Foulsham et al. [26] showed that when participants are simply asked to walk across campus, as they approach other people they avoid looking at their faces and eyes.

While recent real-world investigations have been effective at confirming that there is often a disconnection between life and lab, the goal of cognitive ethology is to bridge this gap. To date, however, there has not been a single study that has set out to apply systematically a cognitive ethological approach to determine if its prescribed methodology will yield data that spans the gap between life and lab.

The aim of the present investigation was to provide precisely such a test. Our first challenge was to decide on what real world phenomenon we should investigate. The idea for a research topic was decided when we referred back to the original cognitive ethology article of Kingstone et al. [19]. This article suggests the following guiding research principles. First, the initial job of the researcher is to observe and describe what people do in the real world in order to specify the domain of inquiry. Such observation should be undertaken in a systematic empirical manner, providing a description of cognition as it operates in real-world settings. Second, the conceptual language used to describe human cognition should, initially, be grounded in the concepts and language that are used by people in their everyday life. Third, studies of human cognition should integrate measures of both objective (third-person) behaviour as well as subjective (first-person) experiences. First-person subjective reports should be combined in a mutually constraining fashion with third-person objective observations of behaviour. It was this final point that suggested to us that an excellent place to start was with the question of how first- and third-person perspectives are represented in real life and in the laboratory.

### Conceptualizing Perspectives

We experience our world from an egocentric (i.e. first-person) perspective and only later develop an ability to understand experiences from the perspective of others (i.e. third-person). Although we exhibit an egocentric bias that persists into adulthood [30], early work in this area has suggested that the ability to infer the perceptual experiences or cognitive state of others develops around the age of 7 [31]. However, others have suggested that non-egocentric perspective-taking can emerge as early as 3–5 years of age [32]–[34]. This ability to distinguish one’s own experiences from the experiences of others is thought to be crucial for the development of self-consciousness (e.g., distinguishing between self experience and others’ experience) and theory of mind (e.g., learning to infer the internal states of others; [35], [36]). Thus, from a relatively young age, distinct representations of first- and third-person perspective appear to emerge. This notion of distinct representations has also been supported by recent neuroimaging evidence. Specifically, adopting either a first- or third-person perspective has been associated with distinct patterns of neural activity [37], [38].

In addition to the interest the concepts of first-person and third-person perspectives have received in the developmental literature, these concepts have also long been employed in studies aimed at furthering our understanding of various aspects of cognition. However, perhaps due to the subjective nature inherent in the notion of first- and third-person perspectives, the issue that arises is that stimuli and task instructions intended to induce perspective-taking have differed substantially between subfields of cognitive psychology. For example, since the seminal work of Nigro and Neisser [39], memory research on perspective has focused on how people view themselves within a recalled scene. McIsaac and Eich [40] have developed a methodology in which participants are instructed to form a mental image of a scene as if seeing it again through their own eyes (first-person field perspective) or as if seeing themselves within the remembered scene, from an external viewpoint of a detached observer (third-person observer perspective). Notably, these instructions, in addition to manipulating viewpoint, emphasize the presence of one’s entire body in the recalled scene, as well as a generalized detachment from the events portrayed.

Neuroimaging studies have proposed different conceptualizations of first- and third-person perspective-taking. For example, Ruby and Decety [41], focusing on motor imagery, presented participants with photographs of familiar objects (as well as auditory sentences describing familiar actions) and instructed them to imagine acting with the object themselves (first-person) or to imagine the experimenter acting with the object (third-person). Unlike the memory investigations, which emphasized different viewpoints by the participant, Ruby and Decety’s version of third-person perspective has participants picturing someone else in the imagined scene, rather than themselves. Thus, the emphasis for this conceptualization of perspective is not viewpoint but the distinction between self and other in terms of physical agency. Vogeley and colleagues [42], [43] also investigated the neural correlates of perspective, utilizing a somewhat more abstract perspective-taking task in which participants were shown a virtual scene consisting of a human-like avatar surrounded by a number of red orbs. In contrast to the person-focused tasks of McIsaac and Eich [40] or Ruby and Decety [41], this task required participants to consider the number of orbs visible from their own perspective (first-person) or from the avatar’s perspective (third-person). Thus, in contrast to an emphasis on viewpoint or agency, the defining feature of third-person perspective in studies such as Vogeley et al.’s is the act of centering multidimensional space on another being.

Recent work has also employed perspective manipulations to understand behaviour that emerges during action observation. For example, Flanagan and Johansson [44] demonstrated that eye movement patterns are similar when performing a block-stacking task (first-person) and when observing the same task being completed by another individual (third-person). Jackson, Meltzoff, and Decety [45] also demonstrated that imitation is facilitated when observing videos taken from ones own perspective (i.e., head-centered, first-person perspective) compared to watching videos of other people. This work presents two additional conceptualizations of first- vs. third-person perspective, the first comparing performing actions vs. observing another’s actions and, the second, comparing the observation of head-centered videos vs. videos of another completing an action. Interestingly, a comparison of performing an action and observing head-centered videos, two conceptualizations of a first-person perspective, also revealed differences in eye movement behaviour and memory performance [46]. Specifically, participants prioritized the selection and subsequent identification of task relevant objects to a greater degree when actually performing the task compared to passively viewing head-centered videos of the task being completed.

### The Problem

While behavioural and neurophysiological investigations diverge in their conceptualization of first- and third-person perspectives, they share a common goal of linking their findings and conceptualization of perspective to how perspective is used in one’s everyday life. For example, McIsaac and Eich [40], introduce the concept of perspective taking by discussing the perspectives people typically assume when recalling various life events, such as their wedding day or what they ate for breakfast that morning. Similarly, despite having participants situated in an fMRI scanner, Ruby and Decety [41] state that their use of auditory stimuli provides an ecologically valid context for investigating differences in first- and third-person perspectives, and Vogeley et al. [43] state that their avatar task taps into perspective as an essential aspect of self-consciousness providing insight into one’s understanding of the relationship between an individual and the objects in the environments. Finally, a clear link to more natural contexts has also been provided by Flanagan and Johansson [44] and Tatler et al. [46] as the first-person perspective conditions employed in their work involved having participants actually perform an everyday task.

Given the multi-faceted lab-based operational definitions of perspective it is reasonable to hypothesize that either some of the lab studies are applying definitions that are not representative of perspective taking in everyday life or conceptualizations of first- and third-person perspectives in everyday life are multi-faceted. An extreme position would be to ask if the third-person perspective is even a meaningful concept in natural terms? If it is not then asking people to conceptualize first- vs. third- person perspectives might be a rather difficult and unnatural task for people to do. In other words, it is unclear whether first- vs. third-person can be represented or captured within an uncontrolled complex situation. Such an outcome would have drastic implications for the ecological validity of the lab-based investigations that employ various conceptualizations of first- and third-person perspectives. Addressing this issue thus seems to be an ideal test case for the cognitive ethology approach.

### Present Investigation

According to the prescribed methodology of the cognitive ethology approach, the goal is to first observe and collect data (both objective behavior and subjective reports) from uncontrolled everyday contexts. This then allows for the generation of hypotheses that can be tested subsequently in the lab. In order to apply this approach to understand how participants’ conceptualize first- and third-person perspectives within their everyday environment, two stages of investigation were required. First, participants were instructed to explore a natural environment and take photographs of scenes that represented their individual interpretation of first- and third-person perspectives. Subjective reports were also collected to combine with the picture-taking task in an attempt to identify key factors in how individuals conceptualize the different perspectives. The purpose of the second stage of the investigation was to assess whether participants’ everyday understanding of first- and third-person perspective generalizes to a lab-based context. To this end, we tested whether the factors identified as important to the understanding of first- and third-person perspectives from the picture-taking task and subjective reports could be used to accurately predict performance in a controlled picture-sorting task. An independent group of participants completed a task where they were instructed to rank order, in the laboratory, a set of images as most-to-least representative of first- or third-person perspective. In short, by grounding the understanding of perspective in the data acquired from a more natural everyday context (Stage 1), a predictive model of participant performance was generated and compared to the participants’ actual performance on the task (Stage 2). It is important to note here that cognitive ethology does not purport to be able to capture in a single study all aspects of human cognition and behaviour that one is interested in understanding, i.e., in the present case first- and third-person perspective. The cognitive ethology approach seeks to acquire an accurate representation of freely occurring natural behaviour and to use that to understand and predict human cognition and behaviour as it occurs within complex real-world situations. Thus by asking participants to take photographs depicting their understanding of first- and third-person perspective, whatever that understanding may be, we are not expecting to capture all factors that affect first- and third-person perspective as it naturally occurs. For example, there may be key features (e.g., action, sound, motion, touch, smell, sound, cross-cultural differences) that are not easily represented by a photograph. The key point is that there is a critical distinction to be drawn between the *method* of cognitive ethology – and its emphasis on reducing experimental control and increasing situational complexity – and the *measurement* of that behaviour that is used and the necessary limitations that this can entail.

## Methods

### Ethics Statement

The present investigation was conducted in accordance with American Psychological Association standards for ethical treatment of subjects and received ethical approval through the University of British Columbia’s Behavioural Research Ethics Board.

### Participants

Sixteen undergraduate students (nine female; ages 18–25 years) were recruited at the University of British Columbia and completed the picture-taking task. An additional eight participants were recruited (4 females, ages 22–60 years) to complete the subsequent picture-sorting task. All participants provided written informed consent and reported normal or corrected-to-normal vision.

### Procedure

#### Stage 1 – Collecting natural behavior in picture-taking task

Upon entering the laboratory, the experimenter and participant engaged in a verbal dialogue regarding the participant’s understanding of the concept of perspective. Importantly, throughout this discussion, the experimenter did not provide a definition of perspective to the participants, thus avoiding any potential bias in how the participants themselves conceptualize first- and third-person perspectives. Once the participant stated that they felt able to complete the picture-taking task, they were loaned a digital camera (Canon Powershot A60) and instructed to take pictures that best represented their understanding of first- or third-person perspective, with the order of perspective counterbalanced across participants. Participants were told that they were free to take as many pictures as they wished from anywhere inside or outside the department of Psychology at the University of British Columbia. Participants were also instructed not to delete any pictures and to return in approximately 10–15 minutes.

Following each picture-taking task, participants were asked a series of questions to gain insight into what key factors they associated with each viewing perspective. Specifically, participants were asked the following three questions: Why did you take the pictures you did? What elements of your picture did you feel needed to be there to best demonstrate what first (or third) person perspective means to you? What picture, of all the pictures you took, do you feel best captures the definition of first (or third) person perspective, and why?

#### Stage 2 – Using everyday behaviour to make predictions in a lab-based picture-sorting task

Participants were presented with 14 photographs and were instructed to order the pictures based on their individual understanding of first- and third-person perspectives. Participants performed the task twice, once arranging the images based on their understanding of first person perspective and again based on their understanding of third person perspective. The order of perspective was counterbalanced across participants.

To control for the possible influence of image valence, the images selected for participants to order were taken from the International Affect Picture System (IAPS). All selected images were ranked as pleasant (ranging in affect from 2.06 to 7.57, with higher values representing more pleasant valence; [47]). Images were also as diverse as possible yet shared similar features and content as the images taken by participants in Experiment 1. For example, some pictures contained people while others were of natural scenes. Pictures containing people encompassed a range of scenarios, with people performing different activities such as standing by a gravestone or playing chess. Some individuals in the pictures also appeared to make eye contact with the camera, while others did not.

The pictures were presented on a computer screen in thumbnail version, measuring 2.2–2.9×2.8–3.0 cm. Each individual picture could be expanded by double-clicking on the thumbnail, with enlarged pictures measuring 20.8–21.1×25.0–30.7 cm. Participants were asked to arrange the pictures left-to-right by dragging and dropping the thumbnail versions of the pictures on the screen with the mouse so that the most representative picture of a given perspective was located in the top left-hand corner of the screen.

## Results

### Picture-taking Task

A total of 274 pictures were taken by participants. Each image was coded based on a categorical scheme derived from the participants’ subjective reports, describing the factors they felt were the most associated with the two perspectives. Specifically, from over 250 collected subjective responses, 27 relatively specific sub-categories were generated (e.g., reports mentioning a “top down angle” were one sub-category). We then grouped these sub-categories into broader categories, each possessing a number of levels. For example, the “top down angle” sub-category was grouped together with three other sub-categories for form the broad “Viewing Angle” factor (see Table 1). This process yielded the following five factors that were critical in distinguishing between perspective conditions and were remarkably consistent across participants, 1) Viewing distance (Near/Middle/Far) 2) Viewing angle (Up/Level/Down) 3) Content (No People/Portrait of face/Portrait with body/Body part) 4) Subject (Self/Other) and 5) Eye direction (Toward camera/Away/Hidden). Each image was categorized across these factors to allow for a comparison of the factors thought to be critical for each perspective. Coding accuracy was validated by an independent researcher not involved in the present study, with two coders agreeing on 96% of the categorizations. A hierarchical log linear analysis (SPSS analysis hiloglinear; SPSS 2010) was conducted combining perspective (first−/third-person) with the 5 factors derived from the subjective reports. Interestingly, an initial sweep of the categorical data revealed that the presence or absence of people in the photos was a factor in all significant interactions. In light of this finding, we formed two broad image categories to compare across participants’ representations of first- and third-person perspectives – pictures that did not contain people (160 pictures, 100 first-person and 60 third-person) and pictures that did contain people (114 pictures, 58 first-person and 56 third-person).

**Table 1:** Coding Scheme Categories Developed From Subjective Reports.

Category	Possible Value	Representative Subjective Report
Distance	Near	“Close up, detail”
	Middle	“What I saw whilst walking around”
	Far	“Pictures taken from further away”
Angle	Tilt up	“Pictures that make me feel likeI am looking up”
	Eye Level	“Pictures taken at eye-level”
	Tilt Down	“Shots looking down”
Content	No people	“Absence of people”
	Portrait of face	“Somebody directly looking at me and smiling”
	Portrait with body	“A picture of someone else engaged in theirown activity that I am not part of”
	Body part	“Looking at my body”
Subject	Self	“Pictures of me doing something”
	Other	“Other people doing things, not me”
	No People	“No people, just an object”
Eye Gaze	Toward Camera	“The more likely there is eye contact,the more likely it is 1^st^ person”
	Away	“Lack of attention to camera”
	Hidden	“Picture of my own body”

#### Pictures without people

**Figure 1:**
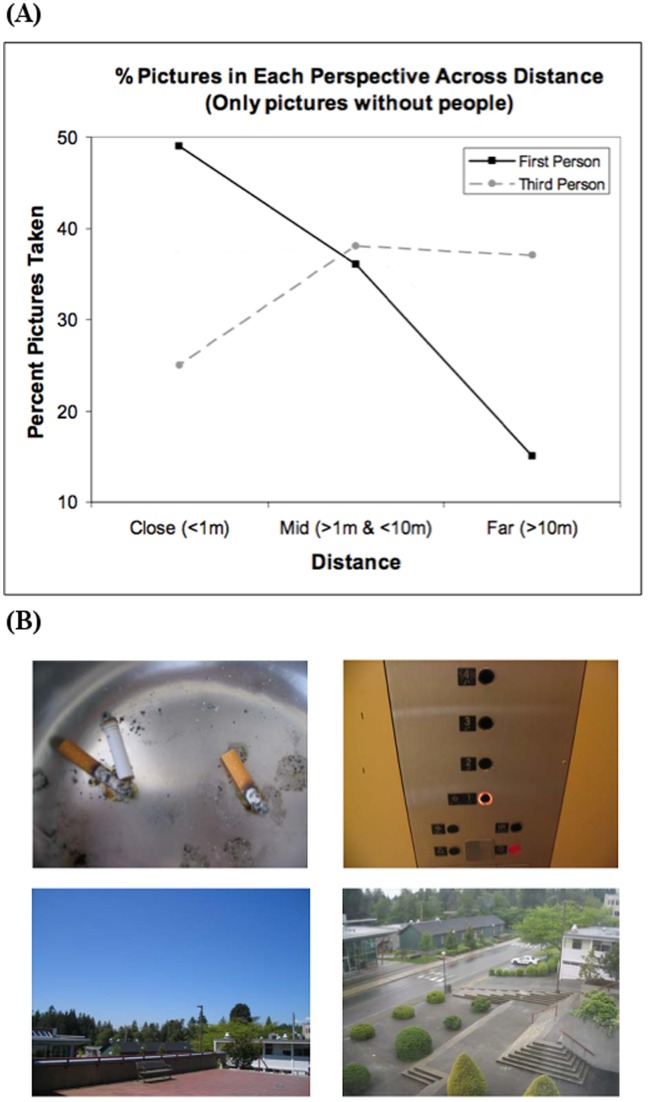
**Figure 1A presents the Perspective × Viewing Distance interaction for pictures without people.** Participants tended to take more close (<1 m) pictures to capture first-person perspective and more far (>10 m) pictures to capture third-person perspective. **Figure 1B provides examples of pictures showing the critical factor of distance in pictures without people.** *Top:* First-person, both pictures demonstrate the tendency to take pictures within personal space. *Bottom*: Third-person, both pictures show subjects representing third-person perspective through distance.

Data for pictures without people were analyzed using a hierarchical log linear analysis ( [48]; SPSS procedure HILOGLINEAR, 2010) with 3 factors, Perspective, Viewing Distance and Viewing Angle. A backward elimination procedure yielded a final model with components Viewing Angle and Perspective × Viewing Distance, i.e., the data are completely accounted for by these two components. The interaction Perspective × Viewing Distance (*χ* ^2^_(2,N = 160)_ = 13.1, p<0.01) is illustrated in Figure 1a and reflects the fact that, to depict a first-person perspective, participants tended to take pictures of objects from a close range (<1 m; 49%) rather than a mid-range (1–10 m; 36%) or a far range (>10 m; 15%). In contrast, to depict a third-person perspective participants tended to take fewer pictures from close range (25%), favoring pictures from mid- and far-ranges (38%, and 37%, respectively). Examples of these photos are shown in Figure 1b. No other effects were significant.

#### Pictures with people

**Figure 2:**
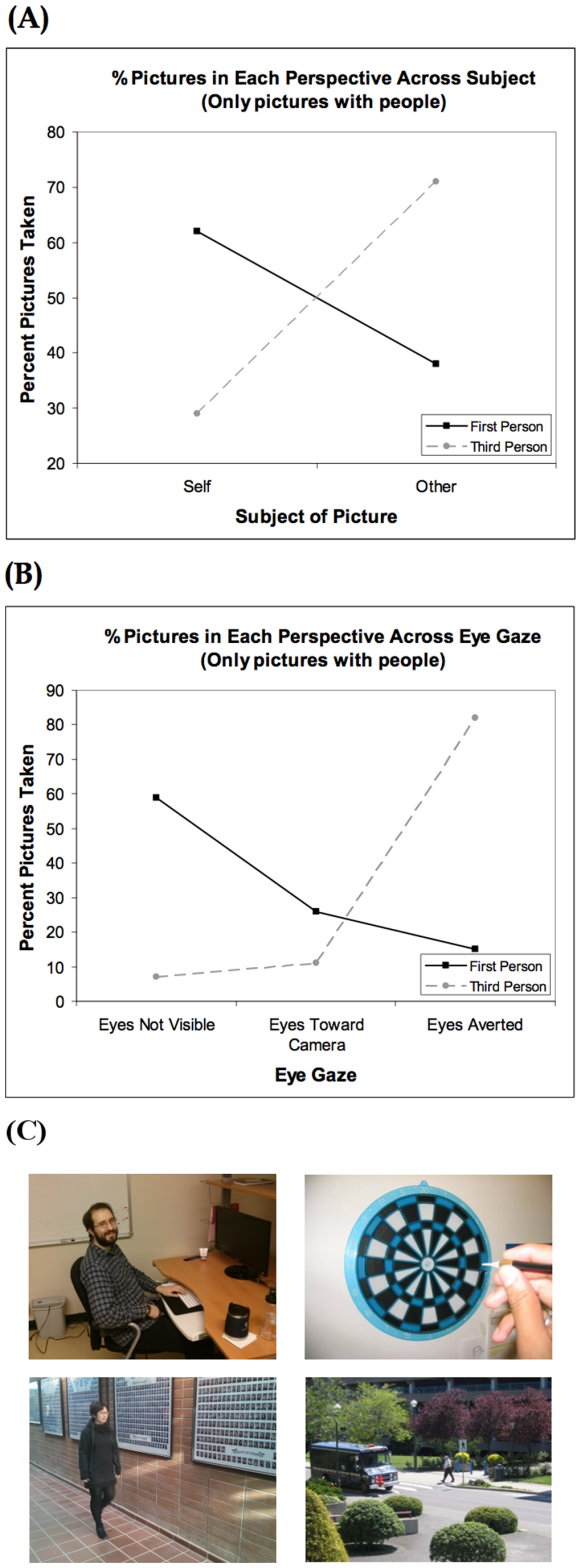
**Figure 2A presents the Perspective × Subject interaction for pictures with people.** Participants took significantly more pictures of themselves (self) in first-person than in third-person. Conversely, subjects usually represented third-person by taking pictures of other people. **Figure 2B presents the Perspective × Eye direction interaction for pictures with people.** First-person perspective was captured by participants by taking pictures either of their own body, or another person who was looking at the camera. Third-person perspective was represented predominantly by taking of pictures of other people who were not looking at the camera. **Figure 2C provides examples of photos showing the Perspective × Subject and Perspective × Eye Direction factors in pictures with people.** *Top left*: First-person, picture of someone else, looking at camera. *Top right*: First-person, participanths picture of their own body performing an action. *Bottom left and right*: Third-person, pictures of other people not looking at camera. Individuals depicted in Figure 2C have given written informed consent (as outlined in PLOS consent form) to publish these case details.

Data for pictures with people were analyzed using a hierarchical log linear analysis with 6 factors - Perspective, Viewing Distance, Viewing Angle, Content, Subject, and Eye Direction. A backward elimination procedure yielded a final model with components Content × Viewing Angle, Content × Eye Direction, Viewing Distance × Eye Direction, Viewing Distance × Viewing Angle, Subject × Viewing Distance, Content × Viewing Distance, Perspective × Subject, Perspective × Eye Direction, and Subject × Eye Direction. In other words, the data are completely accounted for by these six 2-way interactions, and no higher-order interactions are needed to explain the data. In the following, we focus on the two interactions with Perspective. Figure 2a illustrates the significant Perspective × Subject interaction (self vs. other; *χ* ^2^_(1, N = 114)_ = 12.9, *p*<0.01), with participants in the first-person perspective more likely to take pictures of themselves (62%) rather than pictures of other people (38%); whereas, in the third-person perspective, participants were more likely to take pictures of other people (71%) rather than pictures of themselves (29%).

Figure 2b illustrates a significant Perspective × Eye Direction interaction (*χ* ^2^_(2, N = 114)_ = 10.4, *p*<0.01). There are two noteworthy aspects to this interaction. One is that first-and third-person perspectives differed greatly in terms of the percentage of pictures taken with the eyes of the subject hidden (59% vs. 7%, respectively) reflecting the fact that participants in the first-person perspective condition often took pictures of their own bodies that did not involve their eyes being visible. For example, participants often took pictures of one’s own foot or arm from the vantage point of their own eyes to represent a first-person perspective. Second, when the eyes of the person in the photos were visible, in the first-person perspective condition, participants tended to take pictures of people looking toward the camera (26%) rather than away from the camera (15%); whereas, in the third-person perspective, participants tended to take pictures of people looking away from the camera (82%) rather than toward the camera (11%). Finally, it is noteworthy that distance was not significant factor for pictures with people, which stands in contrast to those photos taken without people and described above. Examples of photos representing the reported interactions are shown in Figure 2c.

### Picture-sorting Task

In the above unrestricted picture-taking task, three factors, initially abstracted from the subjective reports, were found to be critical to perspective: viewing distance, subject (self/other), and eye-direction (toward camera/away/hidden). To assess the validity of these factors, we tested whether they could accurately predict performance in a controlled, laboratory-based picture-ordering task. Each picture used in the ordering task were given a +1 score for each first-person factor they contained and a −1 score for each third-person factor they contained, yielding an experimental prediction of each picture’s perspective content. For our limited set of 14 photos, pictures could receive a score ranging from +3 to –3.

**Figure 3:**
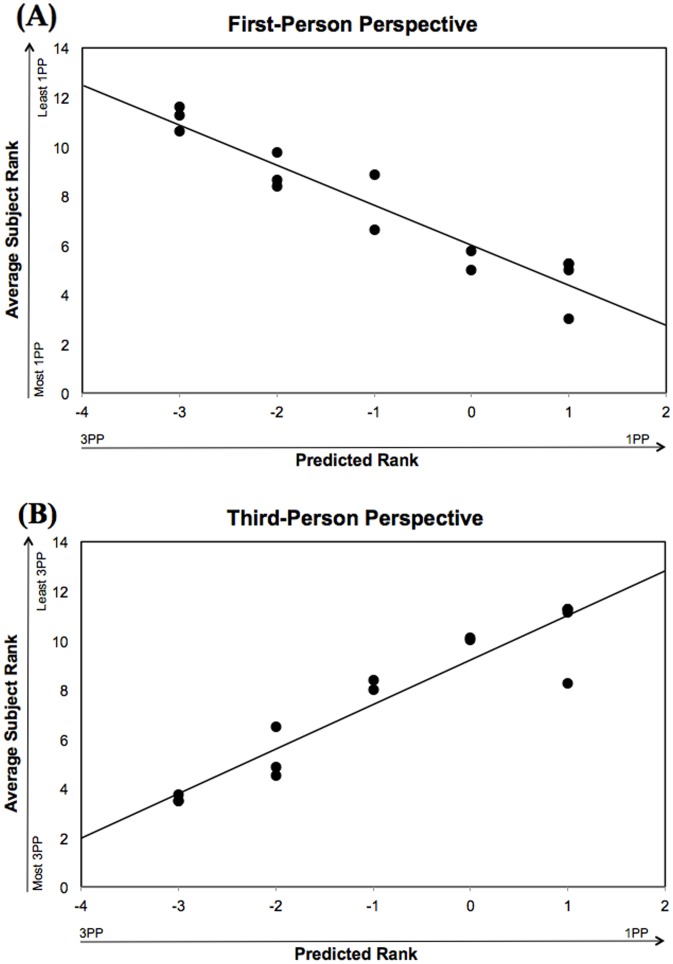
Predicted picture rank based on experimenter scoring scheme compared to average subject rank for each picture in the picture ordering task. (A) Predicted vs. observed for 1PP. (B) Predicted vs. observed for 3PP.

As shown in Figures 3a and 3b, the factors that we obtained from the uncontrolled picture-taking task served as excellent predictors of participants controlled photo ordering performance, thereby validating the method and factors produced by the subjective reports from the picture-taking task. Figure 3a shows the predicted and observed rank-order based on a first-person perspective and Figure 3b shows the predicted and observed rank-order based on a third-person perspective. Note that there is an excellent correspondence between predicted and observed performance for both perspectives, and that the two perspectives appear to be almost mirror functions of one another. These impressions were confirmed statistically, with a correlation analysis of the predicted and observed performance yielding highly significant effects both for the first-person perspective condition, *r* = .95, p<.001, and the third-person perspective condition, *r* = .94, p<.001.

## Discussion

The primary goal of the present investigation was to provide a test of the cognitive ethology approach. The unrestricted picture-taking task coupled with subjective reports provided a way to ground the psychological concepts of first-person and third-person perspective in how people understand them in a natural context. Using these data that were collected without experimental control we then generated predictions of human behavior that were tested subsequently in a lab-based picture-ordering task. The lab-based performance in the picture-ordering task conformed to the predictions we derived from our uncontrolled real-world observations and subjective reports. Collectively, the two stages of our investigation provide a proof of concept for the cognitive ethology approach. Grounding the understanding of perspective in how it is conceptualized in a more natural everyday context revealed that whether a given image was understood as more representative of a first- or third-person perspective depended on both physical (e.g. viewing distance) and social factors (e.g. subject of image, eye-contact). Below we highlight these findings and discuss how they are consistent with previous lab-based conceptualizations yet also reveal a factor that presents a novel contribution to the traditional understanding of perspective taking.

Our results revealed that whether someone was present in the picture or not formed the basis for two broad categories. We found that for pictures without people, distance was the critical factor used to depict first- and third-person perspectives, with photos taken close-up to objects representing a first-person perspective and those taken further away depicting a third-person perspective. This emphasis on viewing distance is consistent with the traditional Nigro and Neisser [39] idea that events recalled from one’s own eyes (i.e. first-person perspective) are thought to have access to more proximal scene representations. This finding also appears to connect with the literature on perspective taking when recalling emotional events. For example, whereas first-person memories tend to contain more references to emotions (e.g., [40], [49]), people tend to adopt a third-person perspective when recalling events that were traumatic [50], physically painful [51], or anxiety-provoking (e.g. [52]–[54]), as it allows for a degree of detachment (i.e. emotional distance) from the negative emotions associated with the recalled events.

In contrast to pictures that have no people present, when pictures contained other people, distance was not a critical factor. Instead the subject within the scene and eye gaze direction emerged as key factors for representing first- and third-person perspective. Many participants took a picture of a portion of their own body to represent first-person perspective. The agency implied by pictures of one’s own body (especially arms and hands) is consistent with Ruby and Decety’s [41] conceptualization, which emphasizes physical agency and imagined motor movements. In addition, these images are also consistent with the investigations that represented a first-person perspective with videos taken from the participants’ perspective (i.e., head-centered perspective, [44]–[46]).

When the participant themselves were not present in the scene, much weight was given to the eyes of the people photographed. If the eyes of those photographed were visible, first- or third-person perspective was determined by gaze direction: in a first-person perspective gaze is directed straight at the participant (camera), and in a third-person perspective gaze is directed away. This finding is novel as, to the best of our knowledge, no previous investigation of perspective taking has considered eye gaze as an important factor. We feel that isolating this social factor is especially important in that the subjective reports of many participants reflected a desire to represent a sense of being involved (first-person) or removed (third-person) from the scene they were depicting. Most notably, participants reported social involvement, captured by direct eye gaze, and social detachment, represented by a lack of attention to the camera by the person in the picture, as being most critical to representing this factor of social engagement. This factor is also reflected in the picture-ordering task. That is, pictures depicting groups of people whose attention was clearly not focused toward the camera were characterized as being predominantly third-person. However, it is worth remembering that our present instantiation of the cognitive ethology approach is not without limitations. For example, the present investigation was drawn from a primarily Western population, thus there exists the potential that this effect is culture-specific. Similarly we still need to apply a cognitive ethology approach with additional measurements (e.g., motion, action, sound) to examine what role other factors play in first- vs. third-person perspective.

### Summary and Conclusion

We have found in the present study that the application of the cognitive ethology approach can simplify and ground complex and sometimes disparate cognitive topics firmly in everyday life, while rendering the issues tractable to laboratory investigation. In the present study we did not preselect the stimuli that we thought would reflect first- and third-person perspective, but rather freed participants to determine those stimuli for themselves in a natural setting. Despite the uncontrolled nature of the task we found that participants gravitated toward a shared understanding of perspective that was robust and reliable and which was defined independent of the researchers’ own understanding. When we selected the images for the picture-sorting study we based our stimulus selection on this everyday conceptualization of perspectives, and were able to accurately predict how people performed on the task. The factors identified as important to the understanding of first- and third-person perspectives were consistent with conceptualizations used in previous work. Thus our results suggest that previous lab-based definitions of first- and third-person perspectives make contact with aspects of how the concepts are understood in everyday settings. Interestingly, our results did, however, reveal a novel social factor not included in traditional conceptualizations of perspective – eye gaze. The present investigation thus highlights the utility of the cognitive ethology approach, when employed in its entirety, to bridge the gap between life and lab.
